# Impact of migration on oral health outcomes of children in multi-beneficial kindergartens in Nanning, Southern China: a cross-sectional study

**DOI:** 10.1186/s12903-023-03212-7

**Published:** 2023-07-21

**Authors:** Nini Xu, Sicheng Deng, Yan Liang, Aihua Chen, Dan Zou, Ling Li, Rongmin Qiu

**Affiliations:** 1 College of Stomatology, Hospital of Stomatology Guangxi Medical University , Guangxi Key Laboratory of Oral and Maxillofacial Rehabilitation and Reconstruction, Guangxi Clinical Research Center forCraniofacial Deformity, Guangxi Health Commission Key Laboratory of Prevention and Treatment for Oral Infectious Diseases, Key Laboratory of Research and Application of Stomatological Equipment, Education Department of Guangxi Zhuang Autonomous Region, Nanning, Guangxi 530021 China; 2 Department of Stomatology, The First Affiliated Hospital of Guangxi University of Chinese Medicine, Nanning, 530002 Guangxi China; 3 Department of Pediatric Dentistry, College & Hospital of Stomatology, Guangxi Medical University, Research Center for Craniofacial Deformity, Guangxi Key Laboratory of Oral and Maxillofacial Surgery Disease Treatment, No.10 Shuangyong Road, Nanning, 530021 Guangxi China

**Keywords:** Dental caries, Multi-beneficial, Migration, Utilization of dental care services

## Abstract

**Objective:**

To explore the effect of children’s migration on their oral health outcomes in multi-beneficial kindergartens in Jiangnan District, Nanning, China, and to provide a basis for improving the oral health of migrant children.

**Methods:**

A cross-sectional study was conducted among 470 children aged 5 years in Jiangnan District, Nanning, Guangxi. A questionnaire was used to collect information on their demographic and socioeconomic background, migration experience, eating habits, oral hygiene behaviours and utilization of dental care services. Dental caries of primary teeth was examined using the decayed, missing, and filled teeth (dmft) index recommended by the World Health Organization. Dental caries experience and oral health-related behaviours were compared between migrant and resident children. The impact of children’s migration attributes on their oral health outcomes was examined by univariate and multivariate analyses.

**Results:**

Among the examined children, 52.3% were migrant children. The prevalence of caries among the children in multi-beneficial kindergartens was 78.3%, and the mean number of dmft was 5.73 ± 5.00. The prevalence of caries was 81.7% for migrant children and 74.6% for resident children (*p* = 0.060). No significant difference was found in the mean numbers of DMFT between migrant children and resident children (5.96 ± 4.81 vs. 5.47 ± 5.20, *p* = 0.139). There were significant differences in the frequency of tooth brushing (*p* = 0.023) and parental help with tooth brushing (*p* = 0.008), typical use of fluoride (*p* = 0.012), regular dental check-ups (*p* = 0.003) and experience of dental fillings for caries (*p* < 0.001) between migrant and resident children. The multivariate logistic regression analysis showed that among the children with caries, the proportion of resident children who had regular dental check-ups was 1.720 times higher than that of migrant children (95% CI = 1.155 ~ 2.560), and resident children were more likely to have caries filled than migrant children (OR = 3.313, 95% CI = 1.585 ~ 6.927).

**Conclusion:**

Oral health status and oral health behaviours were poor among children in multi-beneficial kindergartens in Nanning, China, and migration might be a significant predictive indicator for the poor utilization of dental care services by children. The government departments should make special policy to promote the children’s oral health in multi-beneficial kindergartens, and invest more to cover the migrant children’s utilization of oral health services.

## Introduction

Dental caries is one of the most prevalent diseases among children, and it is the largest global burden on oral health; there are approximately 532 million children plagued by dental caries worldwide [1]. Severe caries of the deciduous teeth not only affects the development of inherited permanent teeth but also greatly harms the maxillofacial and general health of children. Therefore, early prevention and treatment for deciduous dental caries is necessary.

Over the past few decades, China has undergone rapid economic development and urbanization. An increasing number of rural adults have migrated to cities to seek job opportunities and better incomes to support their families. Moreover, some children follow their parents to the cities; as a result, they are called “migrant children”. Generally, migrant children are those under the age of 18 who leave their residences with their parents to live in other places for more than 6 months without changing their household registration (excluding the separation of people and households in municipal areas) [2]. According to the statistics of the Seventh National Census, the internal migrant population in China reached approximately 376 million in 2020, accounting for 27% of the total population, and it increased by 70% over the same period in 2010 [3]. In recent years, with the implementation of the two-child and three-child policies, the number of migrant children has increased yearly, resulting in some social problems that affect all aspects of society. Compared with urban households, migrant population have lower socio-economic status and poorer ability to obtain material and structural conditions, such as food, shelter, secure, employment status, services and amenities. Psychosocial stress, low coping, and anxiety as a result of negative life events and lower social support are unequally distributed to the migrant population and thus contribute to inequalities in oral health [4]. China remains an urban–rural separated household registration system till now, which has negative effects on migrant children’s equal rights in receiving same education and medical treatment as those children registered as urban households [5]. However, at present, scholars at home and abroad mainly pay attention to the educational and mental health issues of migrant children [6–8], whereas few studies have focused on oral health disparities among migrant children.

In China, the local preschool children could receive education in the local public kindergartens, otherwise the migrant children without local household registration have to go to the private kindergartens which require much more payment than the public kindergartens. However, most of the migrant families are relatively poor, they couldn’t afford their children’s preschool education in private kindergarten, and they would leave their kids at home. Therefore, to mainly solve the problem of poor accessibility and affordability of kindergarten for preschool children, especially for those children from lower-income or migrant families, multi-beneficial kindergarten is newly proposed these years. Compared with other types of kindergartens in China, multi-beneficial kindergartens are non-profit preschool educational institutions, and these institutions are run by social organizations, state-owned enterprises, individuals and other social forces, which are encouraged by local governments. These kindergartens are established in accordance with national standards and supported by governmental subsidies, providing public welfare and universal services [9]. As multi-beneficial kindergartens are supported by government subsidies, they are less expensive and parents need to pay less for their children’s preschool education, most migrant children attend such kindergartens. The coverage of multi-beneficial kindergartens in Jiangnan District, Nanning city, Guangxi, southern China, was approximately 65% by November 2019, and the proportion of migrant children was high, even as high as 80% in some kindergartens. It was found that the caries prevalence among 5-year-old migrant children and rural urban migrants was higher than that among resident children in other cities [10], however, as the mainly subjects little is known about the oral health status of migrant children in multi-beneficial kindergartens.

The present study aimed to investigate the impact of migration on the dental caries and oral health-related behaviours of children aged 5 in multi-beneficial kindergartens in Jiangnan District, Nanning. A caries status examination and a questionnaire survey on oral health-related behaviours were conducted. We hypothesized that migrant children in multi-beneficial kindergartens have a higher prevalence of dental caries and poorer oral health-related behaviours compared to resident children living in the same areas.

## Materials and methods

### Study participants

A cross-sectional study was conducted among 470 children aged 5 years in ten multi-beneficial kindergartens from 17^th^ December 2019 to 6^th^ January 2020 in Jiangnan District, Nanning, Guangxi Province in southern China. The sample size was determined based on the formula of as followings:

$$\mathrm{N}={({\mu }_{\alpha } / \delta )}^{2}p (1-p).$$

N is the sample size for the required survey. In this survey, the value of *α* was set to 0.05, *μ*_α_ = 1.96, and *δ* was set to 0.05*p*.

The *p* value was the estimated caries prevalence based on a previously reported of 81.5% among 5-year-old children in Guangxi [11]. Therefore, the calculated minimum sample size was 363 kindergarten children. Moreover, considering the low efficiency of cluster sampling and the loss of follow-up, the sample size was increased by 20%, and therefore, no less than 454 children were required.

To obtain a representative sample size, a two-stage sampling technique was employed. In the first stage, ten kindergartens were randomly selected in Jiangnan District. In the second stage, a cluster sampling method was performed. All of the children aged 5 in the ten selected kindergartens and their parents were invited to participate in this study. Children who did not cooperate with an oral examination were excluded.

The study protocol was approved by the Ethics Committee of Guangxi Medical University (No. 2019–006), and written informed consent was obtained from the parents or guardians of each child in advance.

### Data collection

#### Information on children’s migration experience

In the study, migrant children were defined as children under the age of 18 who had left their residences with their parents to live in the city for more than 6 months without changing their household registration (excluding the separation of people and households in municipal areas) [2]. Two questions related to children’s migration were evaluated: 1) the parents were asked about where their children’s permanent residence was (local household residence or nonlocal household residence); and 2) for the children who were not local household residents, their parents were asked how long their children had left their residences with their parents to live in the city (≤ 2 months, 3–5 months, and ≥ 6 months).

#### Questionnaire survey

A questionnaire was conducted with the parents based on the Fourth Chinese National Oral Health Survey Methods [8], the previous reference [12], and the dental caries risk assessment tool for 0- to 5-year-old children recommended by the American Association of Paediatric Dentists [13]. The questionnaire was structured to collect information on: 1) the participant’s demographic and socioeconomic background, including sex, single-child status, mother’s and father’s education level, and monthly family income; 2) the child’s migration experience; 3) the child’s eating habits, including intake frequency of sugary snacks or beverages at bedtime and sleeping with bottles containing sugary beverages; 4) the child’s oral hygiene behaviours, including the age at which they started tooth brushing, frequency of daily tooth brushing, and parental help with tooth brushing; 5) use of fluoride toothpaste; and 6) utilization of dental care services, including typical use of fluoride and regular dental check-ups.

The questionnaire was pilot tested among 10 parents of 5-year-old children prior to this study. In this study, the participating kindergartens had facilitated the distribution and collection of questionnaires. The children whose parents or guardians did not sign the inform consent or returned the questionnaires were excluded in this study.

### Caries examination

Clinical examinations were conducted by two trained dentists in the classroom under natural light with the children lying on a desk and the examiner seated on a chair behind them. The examiner was trained and calibrated for dental caries diagnosis based on the World Health Organization (WHO) Health Survey Methods for field studies [14]. Caries status was recorded using the decayed, missing, and filled teeth (dmft) index for primary teeth. The intra-examiner and between-examiners calibrations were performed weekly, and the kappa values for numbers of dmft were > 0.85. -

A child who had at least one tooth with caries filled was treated as an individual who had experienced dental caries fillings, which was considered one measurement of the utilization of dental care services.

### Statistical analysis

All data analyses were performed with SPSS statistical software v.25.0 (IBM, Armonk, NY, US). The categorical variables were compared by chi-square test. *T-test* was used to compare the continuous variables distributed normally, and non-parametric test was used for the comparison of continuous variables distributed non-normally.

The main outcome were children’s caries status (caries prevalence and number of dmft), eating habits, oral hygiene behaviours, use of fluoride toothpaste, utilization of dental care services (including typical use of fluoride, regular dental check-ups and experience with dental caries fillings), and parental oral health knowledge and attitude about children’s oral health; the independent variable was migration status (migrant children vs. resident children). First, the distribution of demographic variables, eating habits, oral hygiene behaviours, use of fluoride toothpaste, utilization of dental care services and caries prevalence were compared between migrant and resident children. Second, multivariate logistic regression analyses were used to examine the potential relationship between the children’s migration experience and caries prevalence, eating habits, oral hygiene behaviours, and utilization of dental care services, controlling for demographic and other potential confounding variables. All tests were two-sided, and a *p* < 0.05 was considered significant.

## Results

Among the 578 parents of the selected children, 41 did not return the questionnaires, and 37 returned incomplete questionnaires. Among the 500 children whose parents completed questionnaires, 30 were not local household residents but rather had left their residences with their parents to live in the city for ≤ 6 months; therefore, they were not migrant children. Thus, only 470 children whose parents completed questionnaires were included in further analysis.

Among these 470 children, 246 (52.3%) were migrant children, and 224 (47.7%) were resident children. There were significantly fewer migrant children who were single children than resident children (*p* < 0.001). The education level of resident children’s parents was significantly higher than that of migrant children’s parents (*p* < 0.001). The monthly income (per capita) of migrant families was lower than that of resident families (*p* < 0.001) (Table 1).

**Table 1:** Comparisons of demographic and socioeconomic indicators between migrant and resident children (*N* = 470)

Variables	Total N (%)	Migrant children N (%)	Resident children N (%)	*P value*
Sex				0.194
Boy	256 (54.5	141 (57.3)	115 (51.3)	
Girl	214 (45.5)	105 (42.7)	109 (48.7)	
Single child				0.000
Yes	109 (23.2)	38 (15.4)	71 (31.7)	
No	361(76.8)	208 (84.6)	153 (68.3)	
Mother’s education				0.000
≤ 12 years	303 (64.5)	186 (75.6)	117 (52.2)	
> 12 years	167 (35.5)	60 (24.4)	107 (47.8)	
Father’s education				0.000
≤ 12 years	283 (60.2)	187 (76.0)	96 (42.9)	
> 12 years	187 (39.8)	59 (24.0)	128 (57.1)	
Monthly family income (per capita)				0.007
< 3000 RMB	170 (36.2)	103 (41.9)	67 (29.9)	
≥ 3000 RMB	300 (63.8)	143 (58.1)	157 (70.1)	
Total		246 (52.3%)	224 (47.7)	

More than one-third of the children consumed sugary snacks or sweet beverages frequently. A total of 82.8% of the children reported eating sugary snacks or beverages at bedtime, and 20.0% reported sleeping with bottles containing sugary beverages. Almost one-third of them did not start brushing their teeth until they were 3 years old. Only 29.7% of the children brushed their teeth twice per day; 69.1% of them did not use fluoride toothpaste or did not know what fluoride toothpaste was. Only 14.9% of the children typically used fluoride, and 31.7% had dental check-ups regularly. There were significant differences in the distributions of the frequency of tooth brushing, parental help with tooth brushing, typical use of fluoride and regular dental check-ups between resident and migrant children (*p* < 0.001). Resident children had a higher frequency of tooth brushing, more parental help with tooth brushing, and more typical use of fluoride than migrant children; moreover, migrant children had fewer regular dental check-ups than resident children. Interestingly, there were no significant differences in the intake frequency of sugary snacks, sugary snacks or beverages at bedtime, habits of sleeping with bottles containing sugary beverages, age at which they started to brush their teeth or use of fluoride toothpaste between resident and migrant children (Table 2).

**Table 2:** Comparisons of oral health-related variables between migrant and resident children (*N* = 470)

Variables	Total N (%)	Migrant children N (%)	Resident children N (%)	*p* value
Frequency of sugary snack intake				0.352
≥ Once/day	294 (62.6)	149 (60.6)	145 (64.7)	
< Once/day	176 (37.4)	97 (39.4)	79 (35.3)	
Frequency of sugary snack or beverage intake at bedtime				0.406
Often/occasionally	389 (82.8)	207 (84.1)	182 (81.3)	
Never	81 (17.2)	39 (15.9)	42 (18.8)	
Frequency of sleeping with a bottle containing sugary beverages				0.853
Often/occasionally	94 (20.0)	50 (20.3)	44 (19.6)	
Never	376 (80.0)	196 (79.7)	180 (80.4)	
Age at which tooth brushing started				0.113
< 3 years	238 (50.6)	116 (47.2)	122 (54.5)	
≥ 3 years	232 (49.4)	130 (52.8)	102 (45.5)	
Frequency of tooth brushing				0.023
< Once/day	71 (15.1)	46 (18.7)	25 (11.2)	
≥ Once/day	399 (84.9)	200 (81.3)	199 (88.8)	
Parental help with tooth brushing				0.008
No	77 (16.4)	51 (20.7)	26 (11.6)	
Yes	393 (83.6)	195 (79.3)	198 (88.4)	
Use of fluoride toothpaste				0.075
Yes	145 (30.9)	67 (27.2)	78 (34.8)	
No/unknown	325 (69.1)	179 (72.8)	146 (65.2)	
Typical use of fluoride				0.012
No	400 (85.1)	219 (89.0)	181 (80.8)	
Yes	70 (14.9)	27 (11.0)	43 (19.2)	
Regular dental check-ups				0.003
No	321 (68.3)	183 (74.4)	138 (61.6)	
Yes	149 (31.7)	63 (25.6)	86 (38.4)	
Experience of dental caries fillings^a^ (*N* = 368)				0.000 0.000
No	327 (88.9)	190 (94.5)	137 (82.0)	
Yes	41 (11.1)	11 (5.5)	30 (18.0)	

^a^A child who had at least one dental caries filled was treated as an individual who reported dental caries fillings

Overall, the caries prevalence among the 470 children was 78.3% (*n* = 368), with a mean number of dmft of 5.73 (SD = 5.00). There were no significant differences in the caries prevalence or number of dmft between migrant and resident children (81.7% vs. 74.6%; 5.96 ± 4.81 vs. 5.47 ± 5.20; *p* > 0.05). Among the 368 children with caries, only 11.1% had dental caries filled, and fewer migrant children had dental caries filled than resident children (5.5% vs. 18.0%, *p* < 0.001) (Table 2).

As there were significant differences in the distributions of the frequency of tooth brushing, parental help with tooth brushing, typical use of fluoride, regular dental check-ups and experience with dental caries fillings, further analysis was performed for these variables.

After controlling for demographic and other potential confounding factors, children’s migration status was not associated with their frequency of tooth brushing, typical use of fluoride or parental help with tooth brushing but was significantly correlated with the children’s regular dental check-ups and experience with dental caries fillings (*p* < 0.001). The proportion of resident children who had dental check-ups regularly was 1.720 times higher than that of migrant children (95% CI = 1.155 ~ 2.560), and resident children with caries were more likely to have had caries filled (OR = 3.313, 95% CI = 1.585 ~ 6.927). (Summarized in Tables 3, 4, 5, 6 and 7).

**Table 3:** Summary of logistic regression analysis of the factors related to children’s frequency of tooth brushing per day (*N* = 470)

Variables	Frequency of tooth brushing	
	(< once/day vs. ≥ once/day)^a^	
	Adjusted OR	*p* value
	(95% CI)	
Mother’s education		
≤ 12 years	1.00 (reference)	
> 12 years	1.906 (0.964–3.769)	**0.064**
Monthly family income (per capita)		
< 3000 RMB	1.00 (reference)	
≥ 3000 RMB	1.848 (1.071–3.188)	**0.027**

^a^In the multiple logistic regression analysis, “ < once/day” was set as the reference category

**Table 4:** Summary of logistic regression analysis of the relationship between child’s migration status and parental help with tooth brushing (*N* = 470)

Variables	Regular dental check-ups	
	(No vs. Yes)^a^	
	Adjusted OR	*p* value
	(95% CI)	
Father’s education		
≤ 12 years	1.00 (reference)	
> 12 years	3.227 (1.721–6.049)	**0.000**
Monthly family income (per capita)		
< 3000 RMB	1.00 (reference)	
≥ 3000 RMB	2.213 (1.321–3.707)	**0.003**

^a^In the multiple logistic regression analysis, “No” was set as the reference category

**Table 5:** Summary of logistic regression analysis of the relationship between child’s migration status and typical use of fluoride (*N* = 470)

Variables	Typical use of fluoride	
	(No vs. Yes)^a^	
	Adjusted OR	*p* value
	(95% CI)	
Child’s migration status		
Migrant child	1.00 (reference)	
Resident child	1.624 (0.948–2.783)	**0.077**
Mother’s education		
≤ 12 years	1.00 (reference)	
> 12 years	2.073 (1.220–3.520)	**0.007**

^a^In the multiple logistic regression analysis, “No” was set as the reference category

**Table 6:** Summary of logistic regression analysis of the relationship between child’s migration status and regular dental check-ups (*N* = 470)

Variables	Regular dental check-ups	
	(No vs. Yes)	
	Adjusted OR	*p* value
	(95% CI)	
Child’s migration status		
Migrant child	1.00 (reference)	
Resident child	1.720 (1.155–2.560)	**0.008**
Sex		
Boy	1.00 (reference)	
Girl	1.556 (1.046–2.314)	**0.029**

**Table 7:** Summary of logistic regression analysis of the relationship between child’s migration status and experience of dental caries fillings (*N* = 368)

Variables	Experience of dental caries fillings	
	(No vs. Yes)	
	Adjusted OR	*p* value
	(95% CI)	
Child’s migration status		
Migrant child	1.00 (reference)	
Resident child	3.313 (1.585–6.927)	**0.001**
Monthly family income (per capita)	-	**-**
< 3000 RMB	1.00 (reference)	
≥ 3000 RMB	3.101 (1.246–7.719)	**0.015**

## Discussion

In this survey, the caries prevalence of 5-year-old children was 78.3%, and the mean number of dmft was 5.73, which was higher than that of children of the same age in the fourth National Oral Health Survey in 2015 (71.9%, 4.24) [15]. These results indicated that the caries prevalence was severe in multi-beneficial kindergartens in Jiangnan District, Nanning, Guangxi. It has been found that the caries prevalence among 5-year-old migrant children and rural urban migrants in different cities was higher than that among resident children [16–18], but we did not find any differences in the present study. In this survey, the caries prevalence of migrant children (81.7%) was slightly higher than that of resident children (74.6%), and there was no statistically significant difference. The probable reason might be that we only compared the caries prevalence of migrant children and registered children in multi-beneficial kindergartens. The other migrant children and registered children in non- multi-beneficial kindergartens were not included in this study. Nonetheless, the overall caries prevalence of migrant children in multi-beneficial kindergartens was high, which indicating that we should pay more attention to the oral health status of migrant children.

Oral health behaviours are an important factor that impacts children's oral health, and effective tooth brushing is a good improvement method. The refined hand movements of young children are not very developed, and parental help is needed to improve the efficiency of children’s oral hygiene behaviours, such as tooth brushing [19]. Our present study found that the tooth brushing habits of migrant children were not better than those of resident children and that migrant children received less help from their parents in tooth brushing, which is consistent with the results of Wang [20]. It might that the parents of migrant children are busy making a living, and have a low level of education, resulting in a lack of awareness of their children’s oral health. Our study also demonstrated no significant difference in the frequency of sugary snack intake between the two groups, but more than 60% of the migrant and resident children consumed a sugary snack or beverage more than once a day. Since excessive consuming sweet foods is proved to be a predictive risk indicator of deciduous tooth caries [21–23], actions should be taken to prevent children in multi-beneficial kindergartens from consuming excessive amounts of sugar to promote their oral health.

It has been reported that resident children have better utilization of dental care services [23], and similar results were found in the present study. We also found that migrant children had less typical use of fluoride, fewer regular dental check-ups and caries filled. There might be two probable reasons for this. First, the migrant population has lower health insurance coverage and is not familiar with local oral health service policies, which could stop them access to dental service [17]. Second, the utilization of dental care services is mainly related to family social-economic status [24]. The treatment expense for oral diseases may be a large burden for low-income families, and children from high-income families are more likely to receive treatment for caries [25]. Our study not only showed that migration and family income were associated with children’s experience of dental caries fillings but also showed that the monthly income of migrant children’s families was lower than that of resident children’s families. A survey showed that the dental utilization for children in the past 12 months increased with the annual income of families per capita, the parents’ education level, urban–rural areas, the areas where children lived and dental utilization in the past 12 months are significantly related to the annual income of household per capita [26]. In addition, it was also proved that mothers’ education level was one of the impact factors on the migrant children’s typical use of fluoride while mothers’ education level of migrant children was lower than that of resident children. Mothers are primarily responsible for the care of children. All of these results further demonstrated that migration might be a considerable factor in the utilization of dental care services of children.

Apart from the sites above, it should be point out that, the migrant children follows their parents to the city, the whole family have to accustom themselves to new surroundings, and they must experience stress, cultural adaptation and lack of social support [27, 28], which might influence the migrant children’s oral health behaviours and dental health. But further investigation is warranted.

Unfortunately, in this study, we found that the children’s oral heal behaviours were poor in multi-inclusive kindergartens in Nanning, and they have low dental service use, which resulting high caries prevalence. However, it is cheerful that, the General Office of the National Health and Wellness Commission formulated a national policy in 2017 that dental caries was incorporated into a part of the management of chronic diseases [29], and they also proposed the Healthy Oral Action Plan (2019–2025), in which oral health education, the special action of “reducing sugar intake”, children’s oral health management service, as well as improvement of medical insurance and assistance policies are advocated [30]. According to these policies, the newly-increased funds from the Chinese government are preferentially distributed to the children in poverty-stricken areas to cover the comprehensive interventions of their oral health including regular dental check-ups, pit and fissure sealing and typical use of fluoride. Based on the findings of this survey and combined with China’s policies, several measures could potentially improve the oral health among migrant children. First, we should enhance the public awareness of dental care services with low coverage of migrant children. Secondly, due to socio-economic status, cultural differences and other factors, there are great differences in the demand for dental care services between migrant children and resident children, the government should establish the sound community health care centers and cooperate with relevant hospitals to organize targeted dental care lectures to enhance the oral health awareness of migrant children and their parents. Third, oral health institutions should cooperate with schools to regularly conduct dental care services such as oral examination and typical use of fluoride for migrant children, to improve the utilization of dental care services for migrant children.

This cross-sectional observational study is one of the few studies that focuses on evaluating the effect of children’s migration on their caries and oral health-related behaviours in primary schools in China. This study is also one of our serial studies on migration on left-behind or migrant children’s oral health outcomes, and our previous study showed that parental migration could be a significant risk factor for caries development among 8- to 12-year-old school children in rural China [31]. There were some limitations in this study. First, the data in this study were cross-sectional, which precludes drawing inferences regarding the causal relationships between children’s migration and their oral health outcomes. Further work on this topic should adopt a longitudinal approach. Second, considering that most of the migrant children were in the multi-beneficial kindergartens, we only made a comparison between migrant children and resident children in multi-beneficial kindergartens, and further study should consider children in non-multi-beneficial kindergartens. Third, this study did not evaluate the potential social and emotional impacts experienced by migrant children that may influence their dental health, and future research should consider these aspects to provide a more comprehensive picture of the children's living circumstances and its influence on their oral health. However, based on the sufficient randomly selected sample and multiple regression analyses approach, this study provides new evidence to support the potential association between children’s migration and poor utilization of dental care services and urgent intervention needs for the migrant population.

## Conclusion

In summary, our study provides new evidence that migration could be a predictive indicator of children’s poor utilization of dental care services in multi-beneficial kindergartens in Jiangnan District, Nanning, Guangxi. There is a need for urgent interventions for the migrant population, and the government should advocate policies on promoting oral health education, action of “reducing sugar intake”, oral health management service, and enlarging the coverage of medical insurance and assistance to fully improve the migrant children’s oral health.

## Data Availability

All data generated or analyzed during this study are included in this published article.

## Abbreviations

dmft: Decayed-missing-filled teeth
WHO: World Health Organization
